# Emerging roles of purinergic signaling in anti-cancer therapy resistance

**DOI:** 10.3389/fcell.2022.1006384

**Published:** 2022-09-19

**Authors:** Michele Zanoni, Anna Pegoraro, Elena Adinolfi, Elena De Marchi

**Affiliations:** ^1^ Biosciences Laboratory, IRCCS Istituto Romagnolo per lo Studio dei Tumori (IRST) “Dino Amadori”, Meldola, Italy; ^2^ Department of Medical Sciences, Section of Experimental Medicine, University of Ferrara, Ferrara, Italy

**Keywords:** ATP, adenosine, P2 receptors, cancer therapy resistance, adenosine receptors, CD39, CD73

## Abstract

Cancer is a complex disease with a rapid growing incidence and often characterized by a poor prognosis. Although impressive advances have been made in cancer treatments, resistance to therapy remains a critical obstacle for the improvement of patients outcome. Current treatment approaches as chemo-, radio-, and immuno-therapy deeply affect the tumor microenvironment (TME), inducing an extensive selective pressure on cancer cells through the activation of the immune system, the induction of cell death and the release of inflammatory and damage-associated molecular patterns (DAMPS), including nucleosides (adenosine) and nucleotides (ATP and ADP). To survive in this hostile environment, resistant cells engage a variety of mitigation pathways related to metabolism, DNA repair, stemness, inflammation and resistance to apoptosis. In this context, purinergic signaling exerts a pivotal role being involved in mitochondrial function, stemness, inflammation and cancer development. The activity of ATP and adenosine released in the TME depend upon the repertoire of purinergic P2 and adenosine receptors engaged, as well as, by the expression of ectonucleotidases (CD39 and CD73) on tumor, immune and stromal cells. Besides its well established role in the pathogenesis of several tumors and in host–tumor interaction, purinergic signaling has been recently shown to be profoundly involved in the development of therapy resistance. In this review we summarize the current advances on the role of purinergic signaling in response and resistance to anti-cancer therapies, also describing the translational applications of combining conventional anticancer interventions with therapies targeting purinergic signaling.

## Introduction

Cancer is one of the leading causes of mortality worldwide (Sung et al., 2021), with a rapidly growing incidence. Despite advances in the design and development of effective anticancer therapeutic strategies, resistance to therapy and metastatic dissemination collectively represent crucial obstacles to improving patient survival (Weiss et al., 2022). The emergence of resistant clones results from pre-existing intrinsic factors (Holohan et al., 2013) and/or the activation of adaptive mechanisms (Longley and Johnston, 2005; Marine et al., 2020). Current treatment modalities such as chemo, radiation, target and immuno-therapy increase the levels of metabolic and oxidative stresses, inducing an intense selective pressure on cancer cells mainly through the activation of the immune system and cell death mechanisms (Kroemer et al., 2013, 2022). In addition, such treatments cause profound changes in the tumour ecosystem, promoting the generation of inflammatory and DAMPS, including nucleosides and nucleotides (Martins et al., 2009; Lecciso et al., 2017; Di Virgilio et al., 2018). To adapt to these stressors and survive, cancer, immune and stromal cells engage a wide variety of mitigation pathways related to metabolism, DNA repair, stemness, inflammation and resistance to apoptosis (Galluzzi et al., 2018; Zanoni et al., 2022a; Labrie et al., 2022).

**FIGURE 1:**
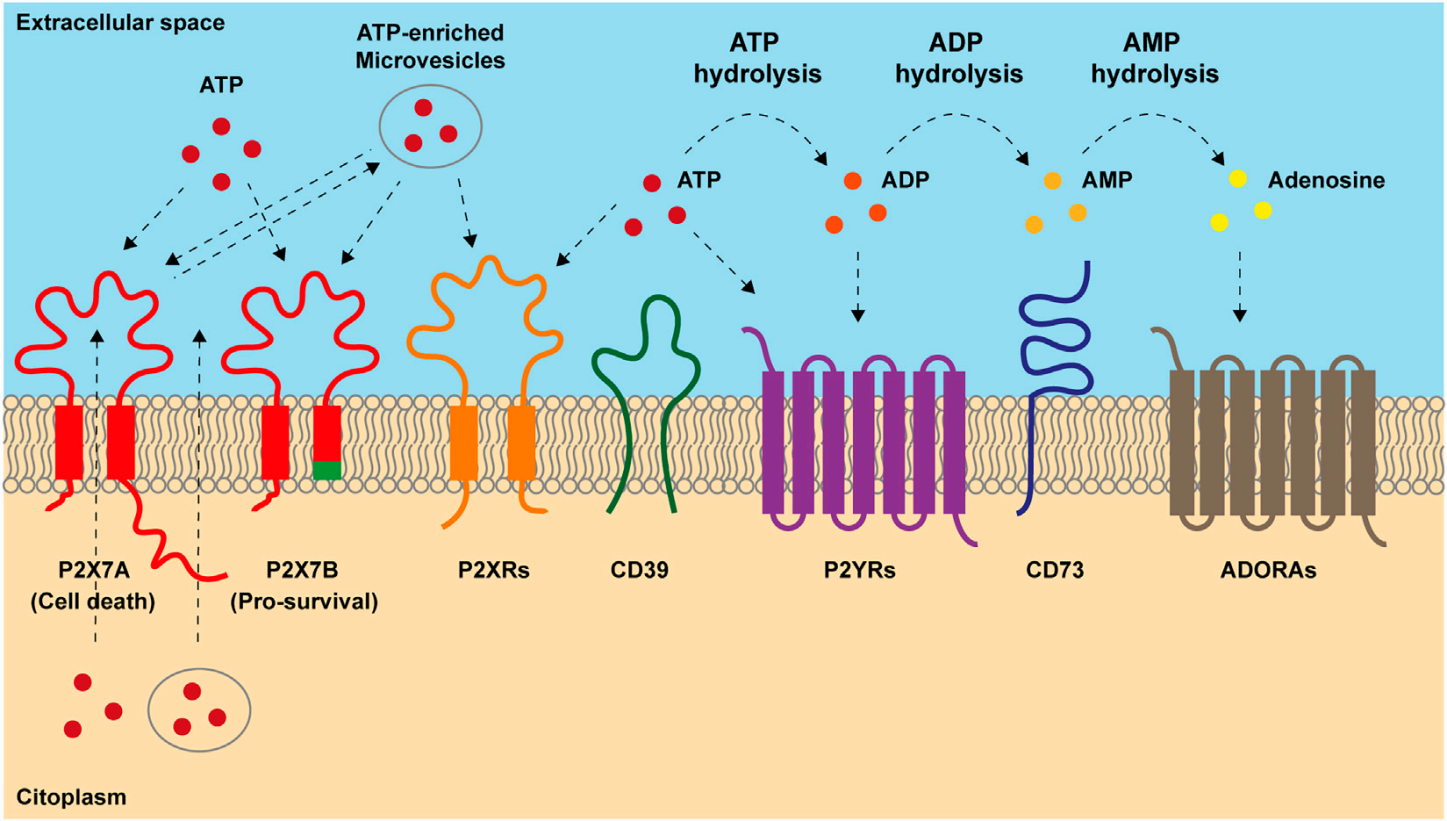
Main components of purinergic signaling. ATP is released into extracellular space both passively, after cellular lysis, and actively, through exocytic vesiscles, exosomes and plasma-membrane derived vesicles or through transporters and channels including the P2X7 receptor. Once accumulated out in the extracellular milieu, ATP acts on P2X and P2Y receptors. P2X7 response to ATP depends on which isoform is engaged. Full-length P2X7A isoform exerts both channel and macropore cytotoxic activities eventually triggering cell death after prolonged stimulation. Truncated P2X7B variant retains only the ion channel activity and does not form the cytotoxic macropore resulting in therapy resitance and tumor growth. In the extracellular milieu, ATP can be hydrolyzed by ectonucleotidases CD39 and CD73 into ADP, AMP and adenosine (ADO). ADP can activate P2Y_12_ receptor and ADO acts on adenosine receptors (ADORAs). ADO can be further hydrolyzed in inosine by adenosine deaminase.

Furthermore, resistant cells often display typical features of cancer progenitor/stem cells, such as high cellular plasticity, increased tumour-initiating and self-renewal abilities, also promoting metabolic reprogramming (Yadav et al., 2020; Jones et al., 2021). Indeed, therapy-induced metabolic switch to mitochondrial- and fatty acids-centred metabolisms leads cancer-resistant clones to a stem-like status. In this undifferentiated form cells can rapidly respond to external cues and promote tumour recurrence and spreading (Marine et al., 2020). Purinergic signaling exerts a pivotal role in these processes as it is involved in the regulation of mitochondrial function (Rabelo et al., 2021; Sarti et al., 2021), stemness (Ratajczak et al., 2020; Rabelo et al., 2021; Fort et al., 2022), cell death (Di Virgilio et al., 2018) and inflammation (Di Virgilio et al., 2017; Huang et al., 2021). The ensemble of reactions activated by extracellular adenosine triphosphate (eATP) and its degradation products ADP, AMP and adenosine (ADO) is known as purinergic signaling. eATP can be recognized by two series of receptors, the P2X ion channels and the P2Y metabotropic receptors. Nevertheless, its primary sensors are P2Xs, as only a few P2Y receptors have eATP as their highest potency ligand (Di Virgilio et al., 2018). In the extracellular milieu, eATP is fastly degraded by ectonucleotidase CD39 in ADP and AMP that is hence transformed into ADO by ectonucleotidase CD73 (Linden et al., 2019) (Figure 1). Adenosine is recognized by P1 receptors, also known as A receptors or ADORA, subdivided into A1, A2A, A2B and A3, which are seven spanning domains metabotropic receptors (Borea et al., 2018) (Figure 1). Thanks to cell death, inflammation and active release, the TME is rich in eATP and ADO (Di Virgilio et al., 2018; Pegoraro et al., 2021b; Vultaggio-Poma et al., 2022) (Figure 1). These purines exert opposite actions on the immune cells as, while eATP is proinflammatory and promotes anti-tumoral immune response, adenosine acts as an immunosuppressant facilitating tumour immune escape (Di Virgilio et al., 2018; Linden et al., 2019). Due to their ATP-degrading/ADO-producing activity, CD39 and CD73 also facilitate tumour progression via immune suppression (Boison and Yegutkin, 2019). However, the effects of both eATP and ADO in the TME are not limited to activity on immune cells as often through P2 and ADORA receptors expressed by either tumour cells or surrounding stroma; they also promote cancer growth, vascularization, and metastasis (Allard et al., 2020; Lara et al., 2020). Here we give an overview of the role played by purinergic signaling in response and resistance to chemo-, radio-, and immuno-therapy, with a particular focus on alternative survival mechanisms adopted by cancer and immune cells. We also describe the translational applications of combining purinergic signalling targeting with conventional anti-tumour therapies.

### Purinergic signaling and chemotherapy resistance

**FIGURE 2:**
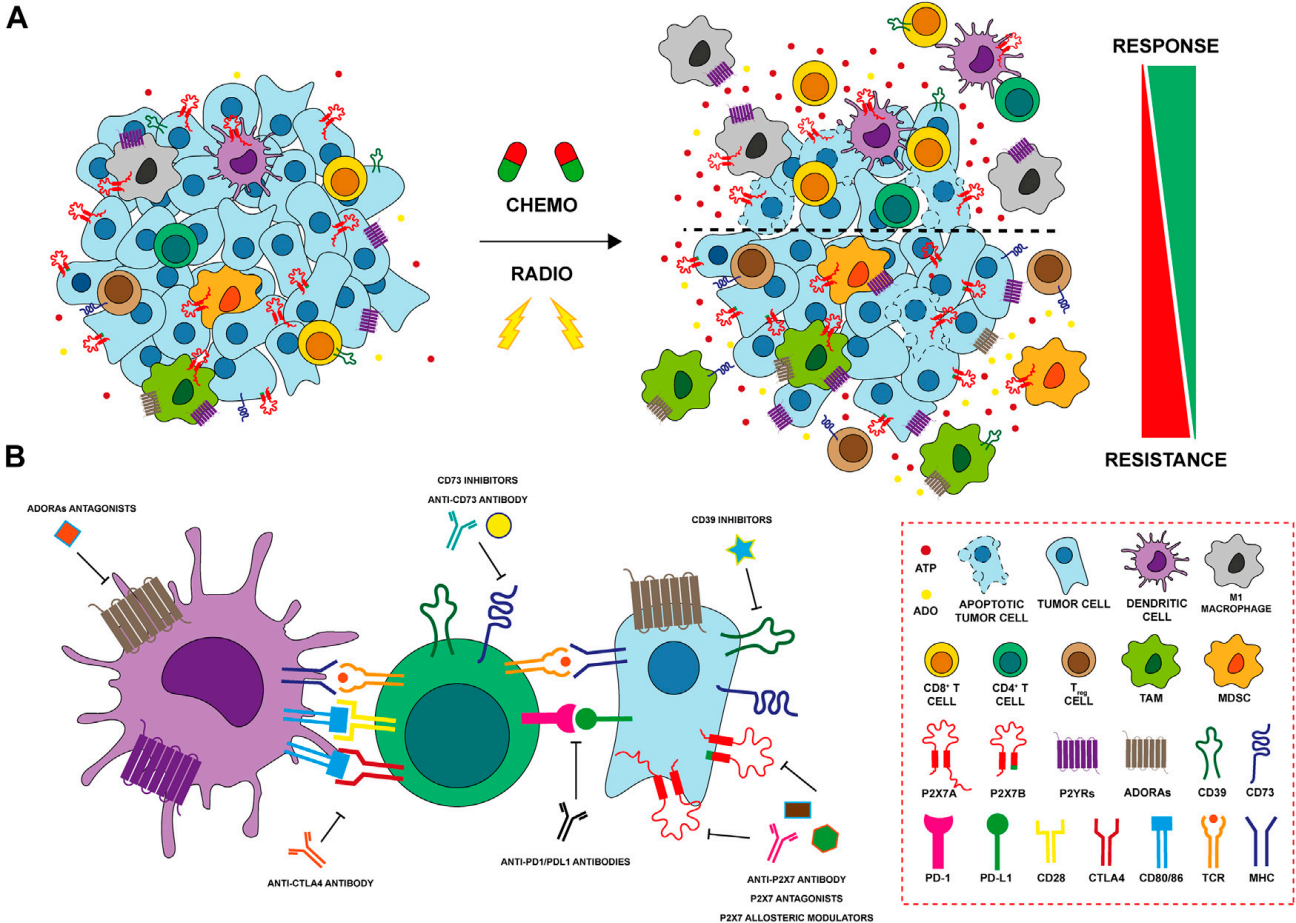
Modulation of purinergic signaling after anti-tumoral therpahy **(A)** Chemo- and radiation therapy (RT) induce cell death and release of ATP in the tumor microenvironment (TME). Cancer cells expressing P2X7A die at a high ATP concentration, while those expressing the truncated P2X7B variant are protected and responsible of tumor recurrence. P2YRs as P2Y_1_, P2Y_2_, P2Y_6_ and P2Y_12_ protect cancer cells conferring resistance to cytotoxic drugs and potentiating cellular response to DNA damage induced by RT. ATP also exerts important effects on immune cells of the TME after therapy. ATP binds P2X and P2Y receptors on macrophages and dendritic cells (DCs) leading to their activation and release of inflammatory cytokines. Activated DCs increase CD4^+^ T helper cells and CD8^+^ T cytotoxic cell responses triggering an anti-tumour immune response. In contrast, increased expression of ectonucleotidases CD39 and CD73 on both cancer and immune cells leads to hydrolization of ATP to ADO, that, in turn, exerts a potent immunosuppressive activity further enhancing the recruitment of T_reg_ and myeloid-derived suppressor cells (MDSCs). ADO activates adenosine receptors (ADORAs) A2A and A2B inhibiting antigen presentation exerted by DCs, promoting M2 macrophage differentiation and impairing CD8^+^ T cytotoxic lymphocyte functions. Finally, both chemotherapy and RT induces the upregulation of ADORAs in resistant tumor cells that, in turn, activate the multiple drug resistance protein-1 (MRP1) and induce stemness and EMT **(B)** P2X7, CD39 and CD73, and ADORAs can be targeted with different therapeutic approaches (*i.e.* using antagonists, allosteric modulators or antibodies) in order to improve antitumor activity affecting both tumor and immune cells in the TME. The combination of immunocheckpoint inhibitors with therapies targeting purinergic signaling represents a promising effective therapeutic strategy in several cancers and it is currently under investigation in preclinical and clinical settings.

Chemotherapy resistance is one of the main issues in cancer treatment. Different mechanisms are involved in drug resistance including DNA damage repair, reduction of chemotherapy entry, suppression of apoptosis, and alteration in drug metabolism (Mansoori et al., 2017). Several studies suggest that the TME can change due to anticancer treatments, influencing cancer cell drug resistance through purinergic signaling. It is well established that the administration of anthracyclines, such as doxo- and daunorubicin, induces the release of ATP from dying tumor cells, activating the immune response via immunogenic cell death (ICD) (Ma et al., 2013; Lecciso et al., 2017) (Figure 2A). A central receptor for ATP in cancer is P2X7 which is expressed and plays a role by both tumor and immune cells. Accordingly, it is also involved in resistance to chemotherapy dependent upon both cell types (Pegoraro et al., 2020; Ruiz-Rodríguez et al., 2020). A recent study on acute myeloid leukemia demonstrated that the cells’ fate after chemotherapy depends on the P2X7 variant expressed by leukemic blasts. Following daunorubicin treatment, leukemic blasts expressing P2X7A, the full form which exerts channel or cytotoxic activities according to stimulation extent (Figure 1), succumb at a high ATP concentration following the opening of a membrane pore. While leukemic blasts expressing the variant B of the receptor, which doesn’t form the pore but retains only the channel function (Figure 1), resist death and proliferate, causing disease relapse. The expression of P2X7B protects the cells from daunorubicin-dependent death and offers an advantage to leukemic blasts in the resistance to chemotherapy (Pegoraro et al., 2020) (Figure 2A). In general, P2X7B variant seems to be central in tumour-promoting activities mediated by the P2X7 among which metastasis (Pegoraro et al., 2021a, 2021b; Tattersall et al., 2021). Also P2Y receptors have been involved in chemotherapy resistance (Woods et al., 2021). For example, P2Y_1_ and P2Y_6_ receptors protect cancer cells from cytotoxic and pro-apoptotic agents (Tan et al., 2019; Placet et al., 2018). Similarly, P2Y_2_ confers resistance to anaplastic lymphoma kinase (ALK) inhibitors (Wilson et al., 2015). Finally, in breast cancer cisplatin treatment upregulates P2Y_12_ expression that sustains the survival of tumor cells counteracting anti-tumoral cisplatin activity (Sarangi et al., 2013). In breast cancer, A2AR expression decreases while P2X7 is upregulated in CD8^+^ T lymphocytes of chemotherapy responders (Ruiz-Rodríguez et al., 2020). Multiple drug resistance associated with adenosine accumulation in the TME was also reported in glioblastoma. Glioblastoma stem-like cells (GSCs) overexpress A3AR that activates PI3K/Akt and MEK/ERK1/2 leading to overexpression and activation of multiple drug resistance protein-1 (MRP1). MRP1 is an ATP-binding cassette transporter that facilitates the efflux of drugs from the cells (Torres et al., 2016). On the contrary, A1R and A2BR promote temozolomide (TMZ) activity in glioma. The combination of TMZ with A1R or A2BR agonists, CHA and BAY606583, has a synergic effect on reducing GSCs’ proliferation. Moreover, the pre-treatment of cells with these agonists before TMZ administration increased and protracted its anti-proliferative effect (Daniele et al., 2014).

### Purinergic signaling and radiation therapy resistance

Radiation therapy (RT), together with surgery and chemotherapy, represents one of the current standard therapeutic options for treating several solid tumours (Larionova et al., 2022). Indeed, more than 50% of cancer patients receive RT for curative and/or palliative purposes (Barker et al., 2015), with substantial improvements in patients’ survival and local tumour control. However, one of the major challenges remains the development of radioresistance mechanisms which leads to worstening in patient outcomes, a higher risk of loco-regional relapse and the development of metastases (Rycaj and Tang, 2014; Tang et al., 2018). RT acts directly by inducing single (SSBs) or double-strand breaks (DSBs) damage to DNA or, indirectly, through the production of reactive oxygen species (ROS) (Zanoni et al., 2019). The resulting oxidative stress can further affect DNA, lipids and proteins structures triggering the activation of stress-response signaling leading to cell death and promoting the release of inflammatory cytokines and the generation of DAMPS (Schaue and McBride, 2010; Barker et al., 2015). DAMPs recognize their corresponding receptors, mediating a radiation damage response that results ICD of tumour cells and in the re-priming of TME immune composition towards an effective anti-tumour immune profile (Krysko et al., 2012). Among DAMPs, ATP can be released or secreted in the extracellular milieu by irradiated damaged cells (Ohshima et al., 2010; Zanoni et al., 2022b), and sensed by P2 receptors (P2Rs) expressed by stromal, immune and cancer cells (Bao and Xie, 2022). eATP accumulation after RT may exert opposite effects on the tumour cells depending on the repertoire of P2Rs and ectonucleotidases expressed. Gehring and others demonstrated that P2X7 is upregulated after radiotherapy in human GBM cells, and its activation leads to cell death due to macropore opening (Gehring et al., 2012). In another study conducted by the same group, P2X7 was confirmed to be essential for RT response *in vivo*, also representing a good prognostic indicator of radiosensitivity in GBM patients (Gehring et al., 2015) (Figure 2A). Nevertheless, relapse occurs in almost all GBM patients also due to the development of RT resistance (Osuka and Van Meir, 2017). In our recent study, we described a novel mechanism underlying radiation resistance involving P2X7 isoforms in GBM. Following RT, GBM cells expressing full-length P2X7A release ATP and are subject to extensive cell death. In post-RT recovery phase, resistant clones undergo a P2X7 isoform switch, characterized by increased expression of the truncated P2X7B variant and the downregulation of P2X7A. Expression of the B variant, coupled with up-regulation of stemness and anti-apoptotic markers, offers a pro-survival, growth promoting advantage to surviving resistant cells. Targeting P2X7 with antagonists in the post RT recovery phase reduces survival of these cells representing a novel potential therapeutic strategy to eradicate RT resistant clones in GBM (Zanoni et al., 2022b) (Figure 2A). Cells that develop radioresistance can increase the DNA damage response (DDR) activating several pathways involved in DNA repair (Tang et al., 2018). In lung cancer, Nishimaki and others have shown that P2X7-dependent ATP release following RT activates P2Y_6_ and P2Y_12_ receptors potentiating the cellular response to DNA damage induced by *γ*-radiation trough EGFR and ERK1/2 activation (Nishimaki et al., 2012; Ide et al., 2014). p53 is the major regulator of DDR mechanisms following genotoxic stresses, like those induced by RT (Li et al., 2012). In normal hematopoietic stem cells, RT increases P2X7 expression in a p53-dependent manner. P2X7 prolonged activation leads to macropore formation, cell death and thus, elimination of damaged irradiated HSC (Tung et al., 2021). However, RT activation of P2X7 can also contribute to hematopoietic dysfunction and eventually lead to the proliferation of leukemic blasts expressing the P2X7B variant. In the immune compartment, eATP engages the P2X7 expressed by dendritic cells activating the NLRP3 inflammasome and promoting the release of IL-1β that, together with antigen presentation, triggers an anti-tumour immune response (Ghiringhelli et al., 2009) (Figure 2A). In addition, eATP can bind the P2Y_2_ receptor on monocytes, recruiting them in the inflamed TME generated by RT (Elliott et al., 2009) (Figure 2A). Accumulation of ATP in TME leads to increased levels of its hydrolytic product ADO via activation of CD39 and CD73. In turn, ADO exerts a potent immunosuppressive activity through its receptors. In the TME of breast cancer RT increases immunosuppressive myeloid cells expressing CD73 (monocytes) and A2AR (granulocytes) (Bansal et al., 2021). In addition, expression of CD73 and of the noncanonical adenosine generation pathway (CD38/CD203a) are upregulated by RT in breast cancer murine and human models, limiting the infiltration of conventional type 1 dendritic cells (cDC1) and consequently activation of CD8^+^ T cells. In suboptimal RT-induced IFN-γ production conditions, CD73 blockade combined with RT enhances cDC1 tumour infiltration, leading to better local and systemic control (abscopal effect) through the induction of an effective anti-tumour T cell response (Wennerberg et al., 2020). In addition, Tsukui and others showed that high expression of CD73 in remnant tumour and stromal cells of surgically resected rectal patients that have received preoperative RT was associated with poor prognosis and increased incidence of recurrence (Tsukui et al., 2020) (Figure 2A). In a gastric cancer model, ADO activates the PI3K/AKT/mTOR pathway through A2AR, leading to increased expression of stemness markers OCT-4, NANOG, SOX-2 and CD44 and finally resulting in RT resistance (Liu et al., 2022). Interestingly, RT resistant breast cancer cells release high levels of extracellular ATP and ADO, also upregulating the expression of A2AR, A2BR, and CD73 (Jin et al., 2021). In this model, ADO produced in the TME promotes EMT, cells invasiveness and lung metastasis activating AKT/β-catenin pathway in a A2AR-dependent manner (Jin et al., 2021) (Figure 2A). Finally, in a murine colon syngeneic model, RT combined with A2AR blockade by DZD2269 antagonist inhibits IR-mediated recruitment of Treg cells restoring T cell function through the expression of IFN-γ (Huang et al., 2020).

### Purinergic signaling and immunotherapy resistance

Immunotherapy, involving reactivation of paused anti-tumoral immune responses, revolutionized oncological treatments offering an efficacious therapy in many previously untreatable cancers. The introduction of checkpoint (programmed death 1/programmed death ligand 1, PD1/PDL1) or cytotoxic T lymphocyte-associated 4 (CTLA-4) inhibitors has substantially increased patients’ life expectancy (Ott et al., 2013; Smyth et al., 2016). However, many side effects related to excessive immune system responses and the high cost of treatments often leads to the discontinuation of immunotherapeutics. Moreover, some patients are non-responsive to these drugs, meaning that alternative combinatorial therapies are needed to obtain better effects. One of the main goals that scholars are trying to achieve is to identify new targets able to enhance antitumor immune responses and, in this context, the role of TME is pivotal. In the last decade, several studies have demonstrated that the adenosinergic system is central in oncology. The combination of approved immunomodulators or therapies that initiate ICD with adenosinergic targeting drugs was shown to increase the efficacy of immunotherapies in preclinical models (Allard et al., 2013; Mittal et al., 2014; Beavis et al., 2015; Young et al., 2016) (Figure 2B), and clinical trials targeting the adenosine pathway in cancer have been launched (Thompson and Powell, 2021). The deficiency of CD73 in CD8^+^ T cells leads to an increase of IFN-γ, TNFα, granzime B production and mitochondrial respiration, meaning that this ectonucleotidase restraints CD8^+^ T cells metabolic fitness (Briceño et al., 2021) (Figure 2B). In melanoma, CD73 is expressed also on exosomes from serum of patients and contributes to lymphocyte T functions suppression and influences the response to anti-PD1 therapy (Turiello et al., 2022). Blocking of both CD73 and A2AR adenosine signaling at the same time, reduces tumor growth and metastasis and improves antitumor immune responses (Young et al., 2016). A2AR antagonism leads also to regeneration of IL-2 and IFN-γ production by T cell and its combination with anti-PDL1 or anti-CTLA-4 treatment improves tumor regression, better than monotherapy (Willingham et al., 2018; Steingold and Hatfield, 2020) (Figure 2B). The first clinical trial, using A2AR antagonist in combination with anti-PDL1 in patients with refractory renal cell cancer, resulted in a clinical benefit associated with an augmented recruitment of CD8^+^ T cells into the tumor and a generation of novel T cell clones in peripheral blood (Fong et al., 2020). Several studies have demonstrated that also blocking the activity of CD39 prevents the synthesis and accumulation of ADO and the consequent generation of an immunosuppressive microenvironment (Beldi et al., 2010; Nikolova et al., 2011; Sun et al., 2013). For example, Perrot and collaborators have generated two antibodies targeting CD39 and CD73 (IPH5201 and IPH5301 respectively) blocking the hydrolysis of ATP into ADO. These antibodies stimulate macrophages and dendritic cells and a recondition/activate T lymphocyte, derived from cancer patients. Moreover, IPH5201 and IPH5301, if combined with immune checkpoint inhibitors or chemotherapy, promote antitumor immune response (Perrot et al., 2019). Clinical trials based on these data have been launched, but no conclusive evidence is available (Augustin et al., 2022; Guo et al., 2022). In cancer cases such as multiple myeloma, where PD1-PDL1 targeting therapy is not generally successful, blockade of CD39, CD73 and ADORA seems to be a valuable immune system reactivating alternative (Yang et al., 2020) (Figure 2B). Noteworthy also P2X7 receptor is involved in dendritic cell-mediated antitumor immune response via activation of the NLRP3 inflammasome (Ghiringhelli et al., 2009; Adinolfi et al., 2019). In line with these data, we have recently demonstrated that tumors growing in P2X7 null mice are characterized by an immunosuppressive TME rich in T regulatory cells overexpressing CD73 and the fitness markers OX40 and PD-1 (De Marchi et al., 2019). Interestingly, these tumors upregulate A2AR, VEGF and TGF-β while reducing anti-tumoral/proinflammatory cytokines (De Marchi et al., 2022). This evidence, together with the finding that in P2X7 null mice TME-ATP levels are reduced (De Marchi et al., 2019), supports the hypothesis that P2X7 deletion causes immunosuppression and neovascularization through increased ADO signaling and A2AR upregulation (De Marchi et al., 2022). In line with these findings, Douget and others have developed a positive allosteric modulator at P2X7, which potentiates the activity of anti PD-1 in lung cancer, leading to tumor regression and the creation of a strong immunological memory (Douguet et al., 2021) (Figure 2B).

## Conclusion

As summarized in this overview, several preclinical studies strongly suggest that combining traditional anticancer interventions with therapies targeting purinergic signaling could be a productive strategy to cure cancer and prevent its relapse. We believe that the field is ripe for translating these findings into the clinical setting by administering chemo-, radio- or immune therapies in combination or sequence with purinergic agonists or antagonists.

## Abbreviations

ADO, Adenosine; ADORA, Adenosine Receptor; ALK, Anaplastic Lymphoma Kinase; BCa, Bladder Cancer; cDC1s, Conventional type 1 Dendritic Cells; CTLA-4, Cytotoxic T lymphocyte Associated 4; DAMPs, Damage Associated Molecular Patterns; DDR, DNA Damage Response; DSBs, Double Strand Breaks; eATP, Extracellular Adenosine Triphosphate; GBM, Glioblastoma; GSCs, Glioblastoma Stem-Like cells; ICD, Immunogenic Cell Death; MRP1, Multiple drug Resistance Protein-1; PD1, Programmed Death protein 1; PDL1, Programmed Death Ligand 1; PRRs, Pattern Recognition Receptors; P2Rs, P2 Receptors; ROS, Reactive Oxygen Species; RT, Radiation Therapy; SSBs, Single Strand Breaks; TME, Tumor Microenvironment; TMZ, Temozolomide; UDP, Uridine Diphosphate.
